# Professor Thomas E. Bond

**Published:** 1872-10

**Authors:** 


### OBITUARY.

---

Professor Thomas E. Bond, M. D.—With sincere sorrow and regret we chronicle the death of the Rev. Dr. Thomas E. Bond, whose name has become so familiar to the dental profession from his long connection with the Baltimore College of Dental Surgery, as Professor of Dental Pathology and Therapeutics. Prof. Bond died of cancer in the stomach, on the 19th of August last, at his country seat, Kalmia, Harford county, Maryland, at the age of fifty-nine years. He was the son of a distinguished divine of the M. E. Church, who in past years was widely known throughout the country as editor of the old *Christian Advocate and Journal*, published in New York city.

The subject of this obituary notice was also a man of great and acknowledged ability, and from his connection with the religious press became as extensively known, and as highly regarded for great talent, strong mind, shining virtues, and large influence, as was his father.

As an editor, he had no superior, having conducted with marked ability the *Episcopal Methodist* and *Baltimore Christian*

*Advocate*, and at the time of his death was associate editor of the *St. Louis Christian Advocate.*

In medicine, as well as theology, Professor Bond acquired considerable eminence, and his loss will be widely felt as that of one who possessed social virtues, integrity, energy, intellect and piety of a high order.

The following notices from the press evince the high estimation in which Professor Bond was held by all who knew him as a writer and as a friend :

* * * "As a writer he had few equals, and, probably, no superiors. His trenchant blade will long be remembered by friends and foes. Few men wielded a pen so facile, a wit so keen, a satire so biting, a sarcasm so withering, or a logic so overwhelming. His articles were sometimes a little acrimonious, yet withal they were attended by a vein of good humor that almost entirely relieved them of unkind asperity. He united to a versatile genius, a brilliant mind, and cogent reasoning powers, an inexhaustible fund of humor and a generous heart."

"By his editorial writings, for many years, he had become widely and favorably know to the reading public of this country, and especially to editorial circles, in which, as a writer, he had but few equals and no superiors. He inherited from an illustrious father great natural abilities, which he had cultivated with painstaking care and industry. In controversy his intellectual qualities were most fully disclosed."

"We think the son exceeded the father in range of thought and in raciness of expression. But his capacity as a writer is known and read of all men. He was well versed in his profession as a physician, and he was a good lecturer from the professional chair ; but he seemed to prefer literary pursuits and ecclesiastical engagements to all others—and he had a special proclivity to editorial work."

"The deceased was a man of strong mind, large attainments, and weighty influence. His colleague of the *Advocate* justly calls this sad event not only a personal grief but a church calamity.

The following is from the pen of one who knew him intimately for many years:

"That Dr. Bond was great everybody knew, but few men knew how good, and pure, and noble he was. He was one of the most fearless of men. He had such a scorn of meanness, and cowardice, and truckling. He was so ready to strike anywhere and at any time that men mistook his fearlessness for belligerence, and supposed him harsh and bitter, but there never was a gentler heart—never a heart freer from malice. In that heart there were no enemies. He loved all men, and though he wielded a battle-axe with a Damascus edge, he never allowed personal feeling to nerve the arm.

"He was sincerity itself. He hated, as I scarcely ever knew men to hate, all cant, and hypocrisy, and pretense. The semblance of it anywhere so aroused him that he may have been unjust in supposing it to be where it was not. He loved truth. He loved Jesus. He loved Christ's people. He fought against their foes everywhere and at all times.

"He was tenderness itself. Weakness, sorrow, melted him at once, and there gushed from his heart a full tide of deepest sympathy. Careful in business, he was never unmindful of the poor and suffering. There is no friend who does not know how free was his purse and how sweet his charity."

Professor Bond graduated in the "University of Maryland, School of Medicine," in his twenty-first year, and practiced medicine in the city of Baltimore for nearly twenty years, during part of which time he held a professorship in the old "Washington University of Medicine."

In 1839, a charter was granted to Horace Hayden, M. D., Chapin A. Harris, M. D., Thos. E. Bond, Jr., M. D., and H. Willis Baxley, M. D., incorporating the "Baltimore College of Dental Surgery," the first and for many years the only Dental College in the world. Professor Bond filled the chair of Dental Pathology and Therapeutics, the duties of which he actively performed up to the time of his death, having survived all of his first colleagues.

In the early history of this College, Prof. Bond, in connection with his professorship filled the position of Dean of the Faculty, being succeeded by the late Prof. Washington R. Handy. Prof. Bond also delivered the Valedictory Address to the first graduating class of this institution, consisting of Drs. Robert Arthur and R. Covington Mackall. More than six hundred alumni hold diplomas bearing his signature, and all living well remember his genial manner in the lecture room, which dullness never entered when he officiated, and will sincerely regret his death.

The Latin diploma of the College was written by Prof. Bond, who was also the author of a work on "Dental Medicine," of which a number of editions have been published, and which has been extensively used as a text book by the profession.

The "Baltimore College of Dental Surgery," has, by this dispensation of Divine Providence, been deprived of its oldest professor, and his loss is sincerely lamented by his colleagues.

On his death bed Professor Bond remarked, "*My work is done,*" but as his colleague of the *Advocate* remarks, "Done on earth, but his name disappearing from the roll of earthly toilers, has been registered in the heavenly places for both consummate reward and new and nobler service."

